# Latent Tuberculosis in Pregnancy: A Systematic Review

**DOI:** 10.1371/journal.pone.0154825

**Published:** 2016-05-05

**Authors:** Isabelle Malhamé, Maxime Cormier, Jordan Sugarman, Kevin Schwartzman

**Affiliations:** 1 Department of Medicine, McGill University, Montreal, Quebec, Canada; 2 Respiratory Epidemiology and Clinical Research Unit, Montreal Chest Institute, Montreal, Quebec, Canada; 3 McGill International Tuberculosis Centre, Montreal, Quebec, Canada; University of Padova, Medical School, ITALY

## Abstract

**Background:**

In countries with low tuberculosis (TB) incidence, immigrants from higher incidence countries represent the major pool of individuals with latent TB infection (LTBI). The antenatal period represents an opportunity for immigrant women to access the medical system, and hence for potential screening and treatment of LTBI. However, such screening and treatment during pregnancy remains controversial.

**Objectives:**

In order to further understand the prevalence, natural history, screening and management of LTBI in pregnancy, we conducted a systematic literature review addressing the screening and treatment of LTBI, in pregnant women without known HIV infection.

**Methods:**

A systematic review of 4 databases (Embase, Embase Classic, Medline, Cochrane Library) covering articles published from January 1^st^ 1980 to April 30^th^ 2014. Articles in English, French or Spanish with relevant information on prevalence, natural history, screening tools, screening strategies and treatment of LTBI during pregnancy were eligible for inclusion. Articles were excluded if (1) Full text was not available (2) they were case series or case studies (3) they focused exclusively on prevalence, diagnosis and treatment of active TB (4) the study population was exclusively HIV-infected.

**Results:**

Of 4,193 titles initially identified, 208 abstracts were eligible for review. Of these, 30 articles qualified for full text review and 22 were retained: 3 cohort studies, 2 case-control studies, and 17 cross-sectional studies. In the USA, the estimated prevalence of LTBI ranged from 14 to 48% in women tested, and tuberculin skin test (TST) positivity was associated with ethnicity. One study suggested that incidence of active TB was significantly increased during the 180 days postpartum (Incidence rate ratio, 1.95 (95% CI 1.24–3.07). There was a high level of adherence with both skin testing (between 90–100%) and chest radiography (93–100%.). In three studies from low incidence settings, concordance between TST and an interferon-gamma release assay was 77, 88 and 91% with kappa values ranging from 0.26 to 0.45. In low incidence settings, an IGRA may be more specific and less sensitive than TST, and results do not appear to be altered by pregnancy. The proportion of women who attended follow-up visits after positive tuberculin tests varied from 14 to 69%, while 5 to 42% of those who attended follow-up visits completed a minimum of 6 months of isoniazid treatment. One study raised the possibility of an association of pregnancy/post-partum state with INH hepatitis (risk ratio 2,5, 95% CI 0.8–8.2) and fatal hepatotoxicity (rate ratio 4.0, 95% CI 0.2–258). One study deemed INH safe during breastfeeding based on peak concentrations in plasma and breast milk after INH administration.

**Conclusion:**

Pregnancy is an opportunity to screen for LTBI. Interferon-gamma release assays are likely comparable to tuberculin skin tests and may be used during pregnancy. Efforts should be made to improve adherence with follow-up and treatment post-partum. Further data are needed with respect to safety and feasibility of antepartum INH therapy, and with respect to alternative treatment regimens.

## Introduction

In 2013, tuberculosis (TB) was responsible for a half a million deaths amongst women worldwide, making TB one of the top killers of women of reproductive age, most being HIV negative[1]. Eleven million Americans, representing 4% of the U.S. general population, are estimated to have latent tuberculosis infection (LTBI) [2]. In countries with low TB incidence, immigrant from higher incidence countries form the major pool of infected individuals[3]. In the United States, the reduction in active TB incidence has in part reflected improvements to screening and treatment of latent infection[3]. Immigrant women face financial, educational and cultural barriers, which can limit health status and health-seeking behaviors. The antenatal period represents an opportunity for them to access the medical system[4]. For this reason, the *American College of Obstetricians and Gynecologists* and the *Center for Disease Control and Prevention* recommends screening all pregnant women at high risk for TB when beginning prenatal care[3, 5]. While treating active disease during pregnancy offers clear benefits, the treatment of LTBI during pregnancy remains controversial and current CDC and ACOG guidelines favor deferring treatment to the post-partum period in most cases[3, 5].

In order to further understand the prevalence, natural history, screening and management of LTBI in pregnancy, we conducted a systematic literature review addressing the screening and treatment of LTBI, in women without known HIV infection. We did not review treatment of latent TB infection in pregnant women with concomitant HIV infection, as the indication for urgent treatment is stronger.

## Materials and Methods

### Information Source and Search Strategy

A librarian from the McGill University Health Center aided in the development of a comprehensive search strategy. Databases searched were Embase, Embase Classic, Medline via PubMed and the Cochrane Library. Articles published from January 1, 1980 to April 30, 2014 were eligible. Search terms used were: pregnancy, or pregnancies, or pregnant, or puerperium, or postpartum, or antepartum, or obstetric, or obstetrical, and mycobacterium tuberculosis, or tuberculosis, or latent tuberculosis.

### Inclusion Criteria

Articles eligible for review were original research publications available online or through inter-library loan. Articles had to be written in English, French or Spanish, the languages spoken by the investigators. Studies included were randomized controlled trials, cohort studies, case-control studies and cross sectional studies. Articles from any country, with relevant information on prevalence, natural history, screening tools, screening strategies and treatment of LTBI during pregnancy were eligible for full review. Articles were excluded if (1) full text was not available (2) articles were written in a language not understood by reviewers (3) they were case series or case studies, meaning case descriptions of pregnant women with latent or active TB, without a comparison group for purposes of analysis; pharmacokinetic studies were considered acceptable. (4) they focused exclusively on prevalence, diagnosis and treatment of active TB (5) the study population was exclusively HIV-infected.

Two independent reviewers (IM, MC) reviewed titles, abstracts, and articles. Titles were screened for relevance to the subject of TB. Any articles reporting original studies with information on LTBI in pregnancy, which did not meet one or more of the exclusion criteria, were retained for full-text review. The investigators independently read full-text versions of eligible articles. Disagreements were resolved by consensus between the two reviewers; where they did not reach consensus, input from a third investigator (KS) was obtained. References from included articles were manually reviewed for additional, potentially eligible articles.

### Data Collection Process

Data abstracted included (1) year of publication; (2) country of study; (3) setting; (4) study design; (5) participant numbers; (6) participant characteristics; (7) recruitment and follow-up period and methods; (8) intervention or exposure; (9) main outcomes or events observed; (10) confounding variables and other covariates considered; (11) main findings.

Participant characteristics described the study population by summarizing eligibility criteria for the study, the method of selection of participants, and their demographic characteristics; when the study design involved matching, the criteria for matching were recorded. Depending on the study question, “Intervention or exposure” represented pregnancy status, treatment administered, testing methods used, TB status, ethnicity, or trimester of pregnancy. Similarly, depending on the study question for each article, main outcomes or events included development of latent or active TB, rates of adherence to testing or to treatment, identification of predictors of active disease, identification of predictors of adherence, and/or correlation of results from different testing modalities. Confounding variables and covariates included age, socioeconomic status, Medicaid coverage, marital status, education, occupation, ethnicity, country of birth, immigration status, language spoken, prior TB screening and treatment, BCG vaccination history, number of antenatal visits, gestational age at first antenatal visit, parity, HIV status, known substance abuse, travel to endemic area, location of residence, exposure to individuals with known TB, and chronic medical conditions.

We included a 3-point quality score (2 = well described, 1 = poorly described, 0 = not described) for the following 8 attributes extracted from the STROBE Statement [6] with an emphasis on methods: (1) description of the study setting; (2) description of study participants; (3) definitions of all variables; (4) description of the data sources and measurement tools; (5) justification of sample size; (6) description of statistical methods; (7) description of results; (8) discussion and interpretation of results. The maximum quality score was therefore 16.

## Results

**Fig 1:**
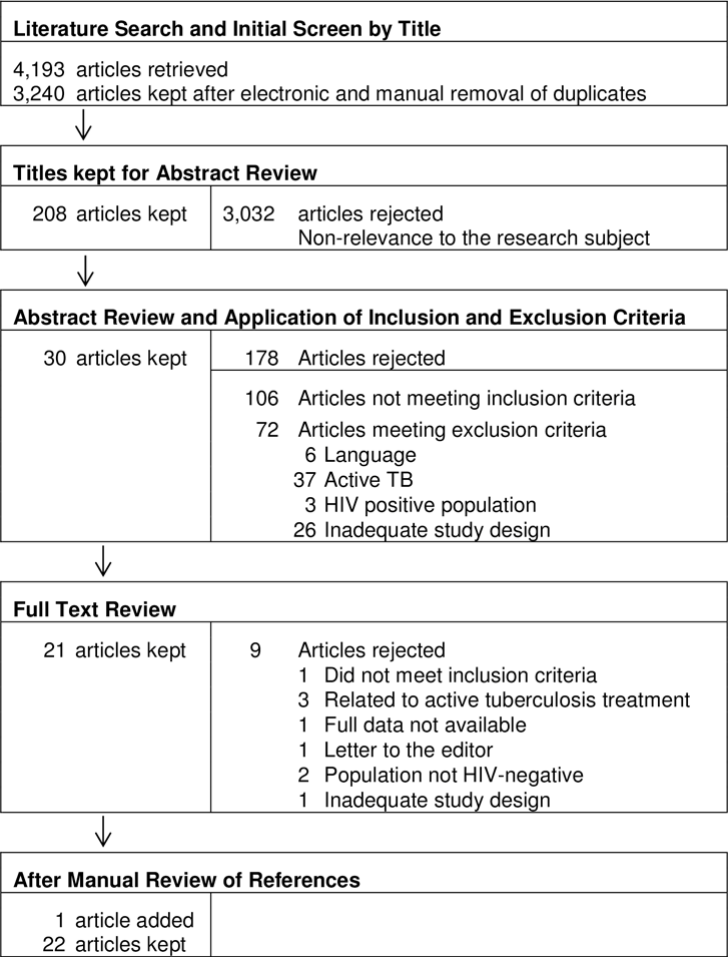
Summary of retrieval and review of articles on tuberculosis and pregnancy, 1980–2014.

The initial search yielded 3, 240 titles, of which 208 titles were retained for abstract review. Of the 208 abstracts reviewed, 30 met inclusion criteria and were eligible. After full text review and manual review of references, 22 articles were retained (Fig 1). Three cohort studies, 2 case-control studies and 17 cross-sectional studies were included. Quality scores ranged from 8 to 15 with a mean quality rating of 12.4 (S1 Appendix). Of these studies, 13 reported prevalence of LTBI during pregnancy; 7 addressed treatment of LTBI pregnancy; 3 addressed the risk of TB reactivation during pregnancy; 5 examined the performance of interferon-gamma release assays during pregnancy, and 6 addressed adherence with TB screening during pregnancy. Some studies investigated more than one of these subjects.

### 1) Prevalence of LTBI

Thirteen studies reported the prevalence of LTBI in cohorts of pregnant women based on tuberculin skin testing (Table 1). In cohorts of pregnant women tested in the USA, the prevalence of latent infection varied from 14 to 48%[4, 7–19]. Skin test positivity was related to the ethnicity of cohort members: In one study 31.3% of Asian-American women, 23.9% of Hispanic women, 9.3% of African-American women and 4.1% of Caucasian women had positive skin tests[4]. Hispanics had a risk ratio of 5.9 (95% CI 3.9 to 8.8) and Asian-American women had a risk ratio of 7.6 (95% CI 3.4 to 17.5) compared with Caucasian women [4]. Of note, 223 of the 1634 skin tests initially placed had to be repeated 2 to 5 times because of failure to return for initial reading[4]. A cross-sectional study conducted in a New York City ambulatory care facility also focused on ethnicity; Asian-American women were again most likely to have a positive tuberculin test with an odds ratio of 3.15 (95% CI 1.62–6.14) relative to white women and 1.55 (95% CI 1.35–1.8) compared to Hispanic women[12]. On multivariate analysis, U.S. born women were substantially less likely to have a positive tuberculin test than were foreign-born women (odds ratio 0.08 95% CI 0.05–0.13)[12].

**Table 1:** Prevalence of LTBI During Pregnancy Measured by Tuberculin Skin Test.

Reference	Country	Study period (y)	Participants (n)	US-born (%)*^1^	Non US-born (%)	Mean age (range)	Interval between testing and reading (h)	Induration cutoff	PPD+ (%)
Cruz [7]	USA	2000	1331	n/s*^2^	n/s	n/s	n/s	n/s	32%
Metersky [8]	USA	1990–1991	1412	n/s	n/s	n/s	before 48	5 mm	18%
							48–72	10 mm	
Magann [9]	USA	n/s	1000	58.5	41.5	25.3 (13–42)	48–72	5–8 mm*^3^	4%
								8–12 mm *^4^	
								12 mm *^5^	
Jackson[10]	USA	2000	30	47	53	27.5	48–72	10 mm	0%
Meints [11]	USA	2003–2006	382	0	100		n/s	n/s	22%
Medchill [4]	USA	1993–1997	1763	38.2	61.8	n/s	48–72	5 mm	15%
Schwartz [12]	USA	2001–2006	4049	11.1	88.9	27.0 (13.0–46)	n/s	10 mm previously positive	48%
Sackoff [13]	USA	1999–2000	558	0	100	26.0 (22–31)	n/s	10 mm previously positive	37%
Lighter-Fisher [15]	USA	n/s	140	59	41	18.5 (13.5–36.5)	48–72	10 mm	20%
Worjoloh [14]	USA	2009–2010	220	35	65	25 (17–41)	48–72	10 mm*^6^	21%
								15 mm	
Chehab [16]	USA		102	10	90	25.9			
Mathad [17]	India	2011–2012	401			n/s	48–72	10 mm	14%
Sheriff [18]	Tanzania	2008	286			25.0 (16–40)	n/s	5 mm *^7^	30%
								10 mm *^8^	
Sepulveda [19]	Chile	n/a	840			n/s	72	10 mm	51–57%

*^1^ When not explicitly detailed, Non-Hispanic Caucasian women were considered US born, whereas women from all other ethnicities were considered non US-born

*^2^ Not specified in article

*^3^ If suspected of having HIV, or close contact with known case

*^4^ If from endemic area, medically underserved low income populations, residents of long term care facility, migrant workers, homeless

*^5^ If no known risk factors

*^6^ Recent immigrants (<5 years), from high prevalence country, injection drug user, residents or employees of high risk congregate settings

*^7^ HIV positive

*^8^ HIV negative

A study from an antenatal care clinic in Northern Tanzania identified members of two tribes as having a lower prevalence of LTBI compared to other clinic patients[18]; these tribes were thought to have better socio-economic status as a result of more fertile lands, and a better knowledge of agricultural techniques [18].

### 2) Natural History: Risk of TB Reactivation with Pregnancy

In order to examine the epidemiology of TB in pregnancy and to establish whether pregnancy is an independent risk factor for active TB, Zenner and colleagues conducted a primary care-based retrospective cohort study, using the General Practitioner Research Database [20]. They considered all women in the UK with known pregnancy start and end dates between 1996 and 2008. The diagnosis of TB was attributed based on culture confirmation, clinical or radiological signs compatible with active TB, or receipt of treatment for active disease. The overall crude incidence rate of active TB diagnosis was 10.1/100,000 (95% CI 8.7–11.8) person-years. The incidence rate for TB during pregnancy was 12.8/100, 00 (95% CI 8–19.4) person-years. The incidence in the same cohort when women were not pregnant was 9.1/100, 000 (95% CI 7.6–10.8) person-years. During the 180-day postpartum period the crude incidence rate was 19.2/100, 000 (95% CI 12–29) person-years. TB occurred significantly more frequently during pregnancy and the 180 days post partum combined, i.e. 15.4/100, 000 (95% CI 11.2–20.6) person-years (crude incidence rate ratio, 1.68 (95% CI 1.17–2.38). After adjustment for age, socioeconomic status, region of residence, and BCG vaccination status, TB incidence was significantly higher during the 180 days postpartum (IRR, 1.95 (95% CI 1.24–3.07) but not during pregnancy itself. However, given the usual time frame over which TB disease evolves, diagnosis post-partum may well reflect the onset of active disease antepartum.

A case-control study from the Dominican Republic did not identify any association between recent pregnancy and TB reactivation [21]. Cases were women with a new diagnosis of active TB, treated at four facilities in Santo Domingo; controls were women who sought HIV screening at the Santo Domingo National Laboratory of Public Health. Case and control subjects had comparable reproductive histories. Among HIV-negative women, those with active TB were no more likely to have been pregnant within the preceding six months than the control subjects (OR 1.1, 95% CI 0.4–2.4). On the other hand, it is not clear whether cases and controls were comparable with respect to other risk factors for active TB, e.g. antecedent smoking and substance use, or what the relative frequency of latent TB infection was in the two groups.

A matched case-control study conducted in a northern province of Malawi, where TB is differentially distributed between men and women depending on age, examined risk factors for active TB among men and women, after adjustment for socioeconomic status and HIV infection [22]. In this study, neither pregnancy nor the post partum period (defined as 9 months after delivery) was associated with active TB. It is possible that in this and other settings, women with incipient active TB, or with associated risk factors, were in fact less likely to become pregnant.

### 3) Screening

#### a) Programs

Studies assessing antenatal screening programs for LTBI and/or active TB revealed a high level of adherence with both skin testing and chest radiography (Table 2) [4, 7, 8, 12–14, 17, 18]. In the USA, reported adherence with antenatal tuberculin skin testing was between 90–100% [4, 7, 8, 12–14] while with chest radiography it was 93–100% [4, 8, 12]. In a study of 4049 pregnant women considered eligible for screening in New York City, Asian-American women were more likely to adhere to testing than Hispanic and Caucasian women[12]. U.S.-born patients were the least adherent with both tests[12]. Of note, this particular screening program did not identify any cases of active TB disease[12]. In higher incidence countries, 71–72% of patients returned for skin test reading, while 100% of pregnant Tanzanian women underwent CXR after it was recommended to them [17, 18].

**Table 2:** Adherence to Antepartum Screening Programs for Latent Tuberculosis.

Reference	Country of origin	Study period (y)	Participants eligible for PPD	PPD placed n (%)	PPD result availablen (%)	Adherence to Chest X-Ray (%)	Participants eligible for treatment evaluation	Adherence to follow-up appointment n (%)	Completion of to >6 months INH n (%)
Cruz [7]	USA	2000	1331	n/s	1195 (90)	n/s	393	167 (42)	71 (42)
Metersky [8]	USA	1990–1991	1412	1405 (99.9)	1405 (100)	254 (98)	272	39 (14)	2 (5)
Medchill [4]	USA	1993–1997	1763	1634 (93)	1497 (92)	211 (93)			
Schwartz [12]	USA	2001–2006	4049	n/s	3847 (95)	1841 (95)			
Sackoff [13]	USA	1999–2000	730	521 (77)	678 (93)		291	202 (69)	27 (13)
Worjoloh [14]	USA	2009–2010	220	220 (100)	199 (95)				
Mathad [17]	India	2011–2012	154	154 (100)	109 (71)				
Sheriff [18]	Tanzania	2008	396	n/s	286 (72)	87 (100)			

#### b) Tools

Four studies compared TST with the Quantiferon-GOLD In-Tube test in pregnant women[14–17]. In three studies from low incidence settings [14–16], concordance between TST and the IGRA was 77, 88 and 91%, with Kappa values of 0.26 (95% CI 0.12–0.40), 0.45 (95% CI 0.26–0.64) and 0.36 respectively. In those studies, the prevalence of TST positivity ranged from 10 to 23%, and IGRA positivity from 5 to 14%. Discordance mostly often reflected TST positive/ IGRA negative results although no specific predictors of discordance were identified. Prior BCG vaccination was a significant predictor of TST positivity [14]. There was no significant difference between stimulated IFN-G levels measured during each trimester of pregnancy [15]. There was no significant difference observed in the IFN-G mitogen-nil response between pregnant and non-pregnant controls of similar age and socio-economic status in an adequately powered analysis [15]. In low incidence settings an IGRA may therefore be more specific and less sensitive than TST in pregnancy, and results do not appear to be altered by pregnancy.

In a study conducted among pregnant women in India, 37% had a positive IGRA in comparison with 14% with a positive TST [17]. Overall agreement was 76% with a kappa of 0.37. The highest frequency of discordance (37%) was observed during the post partum period, and mainly reflected TST negative/ IGRA positive results[17]. On multivariate analysis, being employed and postpartum enrollment were associated with discordance[17]. Education below the 4^th^ grade was associated with a positive TST[17]. Living in an urban setting and being postpartum was associated with a positive IGRA[17]. The median concentration of stimulated interferon-gamma changed significantly with period of pregnancy, with the highest concentration observed in the postpartum period[17]. In a high burden setting, IGRA may be more sensitive than TST. Immune changes during pregnancy, as well as potential repeated exposure, may contribute to the discordance observed, as well as to the increase in the concentration of interferon gamma in the postpartum period.

One study found an association between indeterminate IGRA results and concomitant helminthic infection in pregnant women [23], raising the possibility that helminthic infection may lead to immune alterations that can hamper IGRA testing.

### 4) Treatment

Three studies reported adherence with follow-up of positive tuberculin skin test results, and completion of treatment for LTBI [7, 8, 13]. The proportion of women who attended follow-up after positive tuberculin tests varied from 14 to 69%, while 5 to 42% of those seen after positive skin tests completed at least 6 months of isoniazid treatment [7, 8, 13]. One study found that less than 10% of women potentially eligible for treatment of LTBI completed INH prophylaxis[13]. Attrition was found at every step of the process e.g. lack of referral for evaluation after positive tests, gaps in adherence with follow-up appointments, limited adherence to treatment once prescribed [13]. Another study identified that Asian ethnicity and continuity of care with the same physician in the antepartum and postpartum period were significantly associated with more frequent follow-up and treatment completion rates, while age below 25 was associated with poorer attendance at follow-up appointments [7].

One study examined predictors of INH treatment completion, in Rhode Island; planned treatment initiation during the post partum period was negatively associated with treatment completion [24]. Indeed, 52% of pregnant women referred for postpartum therapy did not return to initiate it [24]. A cross sectional study highlighted that pregnancy was a missed opportunity for screening [25]. Indeed, in a cohort of patients who developed active TB, 22% of missed opportunities for screening in the community arose during pregnancy [25]. Conversely, 40% of patients who were known to have LTBI had been screened during pregnancy[25]. Similarly, the highest proportion of patients with active TB despite previous screening for LTBI were those who failed to initiate INH therapy after testing during pregnancy [25].

After the death of two pregnant women receiving isoniazid treatment in 1982, local health officials requested an analysis of isoniazid hepatitis morbidity and mortality among patients attending a U.S. prenatal clinic, which served a predominantly Hispanic population [26]. A retrospective cohort study compared 3, 681 pregnant and postpartum women enrolled in the 1981 INH preventive therapy program until its termination in 1982, with an unmatched comparison group of 3,948 women aged 15 to 44 years old involved in the 1971 Public Health Service (PHS) multicenter INH hepatitis surveillance study. Two panels of experts determined possible cases of INH hepatitis. Five cases were identified in the prenatal group and 10 cases were identified in the non-pregnant group. Two Hispanic women aged 24 and 27 years old died in the preventive therapy program group at 3 and 5 months postpartum. One death occurred in a 38 year-old non-pregnant black woman in the PHS group. This analysis raised the possibility of associations of pregnancy/post-partum state with INH hepatitis (risk ratio of 2,5, 95% CI 0.8–8.2) and fatal hepatotoxicity (rate ratio 4, 95% CI 0.2–258). However these groups were unmatched, were followed a decade apart, and the analysis was underpowered because of the rarity of these events.

Finally, one cross-sectional study examined the presence of INH in breast milk among lactating women during treatment for LTBI [27]. Peak INH concentrations in plasma and breast milk were measured one hour after administration. Although some isoniazid did penetrate into breast milk, there was considerable inter-individual variability; the calculated mean relative infant dose of 1.2% of weight-adjusted maternal dose was deemed safe [27]. The authors further suggested that lactating women wait at least one-hour interval to breastfeed, after ingesting INH.

## Discussion

LTBI was estimated to be present in up to nearly one half of foreign-born pregnant women tested in the USA. Moreover, the post-partum period may be associated with an increased risk of TB reactivation. In low incidence settings, an IGRA may be more specific and less sensitive than TST, and results do not appear to be altered by pregnancy. Screening programs in pregnant populations revealed excellent adherence with both tuberculin skin testing and CXR. However, adherence with post partum follow-up of positive screening tests was poor, and a minority of women completed treatment. Pregnancy may therefore represent a missed opportunity for treatment of latent infection. There remains some concern about higher incidence of INH-associated hepatitis in pregnancy and the post-partum period. Based on measurements of plasma and breast milk concentration of isoniazid, it is likely safe to administer during lactation.

This study is the first systematic review focusing on LTBI in pregnancy. Using available information from both low- and high- incidence countries, we addressed several aspects of its management, including prevalence, adherence with screening and treatment, new diagnostic tests, and potential treatment toxicity. Most studies were of reasonable quality, with quality rating scores above 10 out of a maximum of 16 points.

Our study was limited by the absence of randomized controlled trials on the treatment of LTBI in pregnancy; we therefore could not draw any firm conclusions about the safety of INH therapy antepartum. There may have been some selection bias with respect to the articles included: we limited our review to studies published after 1980 because we felt that earlier studies would not reflect current practice. Secondly, we excluded 6 articles that were published in languages other than English, French or Spanish. Abstracts were available online for three of those rejected articles: there was one cohort study identifying pregnancy as a risk factor for TB reactivation, one case report and one case series of women with postpartum TB. The titles for each of the other three articles referred to active disease. Finally, we did not review the grey literature. Another limitation was the lack of information concerning the potential use of newer LTBI treatment regimens during pregnancy, e.g. rifampin, combined isoniazid and rifampin, or combined isoniazid and rifapentine [28, 29].

Prevalence of LTBI in HIV seropositive and seronegative pregnant women has been addressed in previous reviews [30, 31], as has adherence to post partum follow-up of LTBI [31]. However, we were able to retrieve studies not included in those earlier reviews. Hence, while not all authors have concluded that pregnancy affects the evolution of latent TB infection [32], we believe that pregnancy may be a minor risk factor for disease reactivation whether it is through the immune changes of pregnancy itself, or the immune reconstitution that follows delivery [20]. In addition, post partum TB may involve more severe disease, including immune reconstitution inflammatory syndrome (IRIS) and a high mortality rate. A case-series of 29 cases of postpartum TB published in 2003 [33] described 93% of women with extra-pulmonary disease, and 69% with CNS disease, although this series may have reflected publication bias. In those cases, treatment was initiated at a median 27 days after the onset of symptoms, and the mortality rate was 38%[33].

A Markov decision analysis model estimated the cost effectiveness of antepartum or postpartum treatment of LTBI with 6 months of INH therapy [34]. With an assumed 90% adherence to post-partum follow-up of a positive tuberculin test, and an assumed mortality rate of 0.001% related to INH-induced hepatitis, treatment initiated at 20 weeks of gestation was estimated to result in the fewest cases of TB [34]. It was predicted to be less costly than postpartum treatment or no treatment [34]. Our review suggests substantially lower adherence to post-partum follow-up for latent TB infection, which may favor antepartum treatment with regards to cost-effectiveness.

The frequently cited study suggesting pregnancy as a risk factor for INH induced hepatitis and associated mortality had wide confidence intervals which crossed the null value of 1 [26]. It used unmatched historical controls for comparison, while assessment of risk factors for INH induced hepatitis (e.g. alcohol consumption, baseline elevation of transaminases and viral hepatitis screen) was not consistent [26]. Close monitoring of liver function tests during antepartum therapy with isoniazid could decrease the rate of clinically significant hepatitis. In view of these findings, it appears clinically relevant to consider further investigation of antepartum treatment for LTBI, particularly in the setting of other risk factors for reactivation e.g. diabetes. It would also be highly relevant to collate and publish outcomes of such treatment, e.g. through cooperative registries or networks such as the Tuberculosis Epidemiologic Studies Consortium (TBESC)[35]. An ongoing clinical trial on treatment of LTBI during pregnancy in HIV infected women, may allow for new insight into the previously described potential increased risk of hepatitis [36].

In addition, since current practice continues to emphasize deferring treatment of LTBI until after pregnancy, it would be important to examine strategies to enhance adherence among women who are treatment candidates.

## Conclusion

For women at risk, pregnancy provides an important opportunity to screen for latent TB infection. As women are already in care, adherence with tuberculin testing and chest radiography is high when these tests are recommended. However, adherence to post-partum follow-up and treatment is much lower, making pregnancy a missed opportunity for treatment of latent infection. Interferon-gamma release assays may be considered as useful alternatives to the tuberculin skin test. The evidence base documenting treatment toxicity during pregnancy is limited, making further research in this area highly relevant.

## Supporting Information

S1 Appendix Grading the Evidence. (DOCX)

S2 Appendix PRISMA Checklist. (DOCX)
