# Supplementary material for: Latent Tuberculosis in Pregnancy: A Systematic Review
Source: PLoS One. 2016 May 5;11(5):e0154825. doi: 10.1371/journal.pone.0154825 (PMC4858222; doi:10.1371/journal.pone.0154825)
Supplement: S1 Appendix — (DOCX) [file pone.0154825.s001.docx]

**Appendix 1: Grading the Evidence**

| **Study Question** | **Article** | **Setting** | **Participants** | **Variables** | **Data Sources measurements** | **Study Size** | **Statistical Methods** | **Results** | **Discussion** | **Total** |
| --- | --- | --- | --- | --- | --- | --- | --- | --- | --- | --- |
| Anergy and TST during pregnancy | Jackson et al [1] | 2 | 2 | 1 | 2 | 2 | 2 | 2 | 1 | 14 |
| Compliance with LTBI screening during pregnancy | Metersky et al [2] | 1 | 2 | 1 | 1 | 1 | 0 | 1 | 1 | 8 |
| Prevalence of LTBI during pregnancy, and compliance with screening | Kwara et al [3] | 2 | 2 | 2 | 2 | 1 | 2 | 2 | 2 | 15 |
|  | Schwartz et al [4] | 2 | 2 | 2 | 1 | 1 | 2 | 2 | 1 | 13 |
|  | Scheriff et al [5] | 2 | 2 | 1 | 1 | 2 | 2 | 1 | 2 | 13 |
| Prevalence of LTBI during pregnancy | Medchill et al [6] | 2 | 2 | 1 | 1 | 1 | 2 | 2 | 1 | 12 |
|  | Magann et al [7] | 1 | 1 | 1 | 1 | 1 | 2 | 1 | 1 | 9 |
|  | Meints et al [8] | 2 | 2 | 2 | 2 | 0 | 2 | 2 | 2 | 14 |
| Prevalence of LTBI, effect of BCG vaccination during pregnancy | Sepulveda et al [9] | 1 | 2 | 2 | 2 | 1 | 2 | 2 | 1 | 13 |
| Compliance with screening, treatment of LTBI during pregnancy | Slopen et al [10] | 2 | 2 | 2 | 2 | 1 | 2 | 2 | 2 | 15 |
| Prevalence, compliance with screening, treatment of LTBI during pregnancy | Sackoff et al [11] | 2 | 2 | 1 | 1 | 1 | 1 | 2 | 2 | 12 |
| Risk of TB reactivation during pregnancy | Zenner et al [12] | 2 | 2 | 1 | 1 | 2 | 2 | 2 | 2 | 14 |
|  | Crampin et al [13] | 1 | 2 | 2 | 2 | 1 | 1 | 2 | 1 | 12 |
|  | Espinal et al [14] | 2 | 2 | 2 | 2 | 1 | 2 | 1 | 1 | 13 |
| Interferon-gamma release assay testing during pregnancy | Chehab et al [15] | 1 | 2 | 1 | 2 | 0 | 1 | 1 | 1 | 9 |
|  | Lighter-Fisher et al [16] | 1 | 1 | 2 | 2 | 2 | 2 | 2 | 1 | 13 |
|  | Worjoloh et al [17] | 2 | 2 | 2 | 2 | 2 | 2 | 2 | 1 | 15 |
|  | Mathad et al [18] | 2 | 2 | 2 | 2 | 2 | 2 | 2 | 1 | 15 |
|  | Gebreegziabihe et al [19] | 2 | 2 | 1 | 1 | 1 | 2 | 1 | 1 | 11 |
| Post partum compliance with treatment | Cruz et al [20] | 1 | 2 | 2 | 2 | 1 | 2 | 2 | 1 | 13 |
| Isoniazid safety during pregnancy | Franks et al [21] | 1 | 1 | 1 | 2 | 1 | 2 | 2 | 1 | 11 |
| Isoniazid safety in breastfeeding mothers and their infants | Singh et al [22] | 0 | 0 | 2 | 2 | 0 | 2 | 2 | 1 | 9 |

References

1. Jackson TD, Murtha AP. Anergy during pregnancy. Am J Obstet Gynecol. 2001;184(6):1090-2. PubMed PMID: 2001176584.

2. Metersky ML, Catanzaro A. A rapid tuberculosis screening program for new mothers who have had no prenatal care. Chest. 1993;103(2):364-9. Epub 1993/02/01. PubMed PMID: 8432120.

3. Kwara A, Herold JS, Machan JT, Carter EJ. Factors associated with failure to complete isoniazid treatment for latent tuberculosis infection in Rhode Island. Chest. 2008;133(4):862-8. PubMed PMID: 2008185484.

4. Schwartz N, Wagner SA, Keeler SM, Mierlak J, Seubert DE, Caughey AB. Universal tuberculosis screening in pregnancy. Am J Perinatol. 2009;26(6):447-52. PubMed PMID: 2009313757.

5. Sheriff FG, Manji KP, Manji MP, Chagani MM, Mpembeni RM, Jusabani AM, et al. Latent tuberculosis among pregnant mothers in a resource poor setting in Northern Tanzania: A cross-sectional study. BMC Infect Dis. 2010;10(52). PubMed PMID: 2010236790.

6. Medchill MT, Shy KK, Johnson AI, Schwartz ML, Plaut M. Prenatal purified protein derivative skin testing in a teaching clinic with a large Hispanic population. Am J Obstet Gynecol.1999;180(6 I):1579-83. PubMed PMID: 1999221418.

7. Magann EF, Leininger WM, Whitworth NS, Klausen JH, Morrison JC. Prevalence of positive PPD reactions and patients' ability to interpret results. Prim Care Update Ob Gyns. 1996;3(4):143-5. PubMed PMID: 1996217365.

8. Meints L, Chescheir N. Screening for infectious diseases in pregnant, foreign-born women from multiple global areas. J Reprod Med. 2010;55(10):382-6. PubMed PMID: 2010686539.

9. Sepulveda RL, Gonzalez B, Gerszencveig R, Ferrer X, Martinez B, Soreasen RU. The influence of BCG immunization on tuberculin reactivity in healthy Chilean women in the third trimester of pregnancy. Tuber Lung Dis. 1995;76(1):28-34. PubMed PMID: 1995067176.

10. Slopen ME, Laraque F, Piatek AS, Ahuja SD. Missed opportunities for tuberculosis prevention in New York City, 2003. J Public Health Manage Pract. 2011;17(5):421-6. PubMed PMID: 21788779.

11. Sackoff JE, Pfeiffer MR, Driver CR, Streett LS, Munsiff SS, DeHovitz JA. Tuberculosis prevention for non-US-born pregnant women. Am J Obstet Gynecol. 2006;194(2):451-6. PubMed PMID: 2006063341.

12. Zenner D, Kruijshaar ME, Andrews N, Abubakar I. Risk of tuberculosis in pregnancy: A national, primary care-based cohort and self-controlled case series study. Am J Respir Crit Care Med. 2012;185(7):779-84. PubMed PMID: 2012239060.

13. Crampin AC, Glynn JR, Floyd S, Malema SS, Mwinuka VK, Ngwira BM, et al. Tuberculosis and gender: exploring the patterns in a case control study in Malawi. Int J Tuberc Lung Dis. 2004;8(2):194-203. Epub 2004/05/14. PubMed PMID: 15139448.

14. Espinal MA, Reingold AL, Lavandera M. Effect of pregnancy on the risk of developing active tuberculosis. J Infect Dis. 1996;173(2):488-91. PubMed PMID: 1996046250.

15. Chehab BM, Kallail KJ, El Fakih RO, Zackula RE, Minns GO. Use of the QuantiFERON®-TB Gold Assay in pregnant patients. KJM. 2010;3(2):24-30.

16. Lighter-Fisher J, Surette AM. Performance of an interferon-gamma release assay to diagnose latent tuberculosis infection during pregnancy. Obstet Gynecol. 2012;119(6):1088-95. PubMed PMID: 2012311855.

17. Worjoloh A, Kato-Maeda M, Osmond D, Freyre R, Aziz N, Cohan D. Interferon gamma release assay compared with the tuberculin skin test for latent tuberculosis detection in pregnancy. Obstet Gynecol. 2011;118(6):1363-70. PubMed PMID: 2011643422.

18. Mathad JS, Bhosale R, Sangar V, Mave V, Gupte N, Kanade S, et al. Pregnancy differentially impacts performance of latent tuberculosis diagnostics in a high-burden setting. PLoS ONE. 2014;9(3). PubMed PMID: 2014284004.

19. Gebreegziabiher D, Desta K, Howe R, Abebe M. Helminth infection increases the probability of indeterminate quantiferon gold in tube results in pregnant women. BioMed Research International. 2014;2014(364137). PubMed PMID: 2014184245.

20. Cruz CA, Caughey AB, Jasmer R. Postpartum follow-up of a positive purified protein derivative (PPD) among an indigent population. Am J Obstet Gynecol. 2005;192(5):1455-7. PubMed PMID: 15902136.

21. Franks AL, Binkin NJ, Snider DE, Jr., Rokaw WM, Becker S. Isoniazid hepatitis among pregnant and postpartum Hispanic patients. Public health reports (Washington, DC : 1974). 1989;104(2):151-5. Epub 1989/03/01. PubMed PMID: 2495549; PubMed Central PMCID: PMCPmc1580026.

22. Singh N, Golani A, Patel Z, Maitra A. Transfer of isoniazid from circulation to breast milk in lactating women on chronic therapy for tuberculosis. Br J Clin Pharmacol. 2008;65(3):418-22. Epub 2007/12/21. doi: 10.1111/j.1365-2125.2007.03061.x. PubMed PMID: 18093257; PubMed Central PMCID: PMCPmc2291261.
